# Hepatitis C virus infection and related liver disease: the quest for the best animal model

**DOI:** 10.3389/fmicb.2013.00212

**Published:** 2013-07-26

**Authors:** Laurent Mailly, Eric Robinet, Philip Meuleman, Thomas F. Baumert, Mirjam B. Zeisel

**Affiliations:** ^1^Inserm U1110, Université de StrasbourgStrasbourg, France; ^2^Institut Hospitalo-Universitaire de StrasbourgStrasbourg, France; ^3^Center for Vaccinology, Ghent University and HospitalGhent, Belgium; ^4^Pôle Hépato-digestif, Hôpitaux Universitaires de StrasbourgStrasbourg, France

**Keywords:** hepatitis C virus, liver disease, hepatocellular carcinoma, animal models, antivirals, immunocompetent mouse model

## Abstract

Hepatitis C virus (HCV) is a major cause of cirrhosis and hepatocellular carcinoma (HCC) making the virus the most common cause of liver failure and transplantation. HCV is estimated to chronically affect 130 million individuals and to lead to more than 350,000 deaths per year worldwide. A vaccine is currently not available. The recently developed direct acting antivirals (DAAs) have markedly increased the efficacy of the standard of care but are not efficient enough to completely cure all chronically infected patients and their toxicity limits their use in patients with advanced liver disease, co-morbidity or transplant recipients. Because of the host restriction, which is limited to humans and non-human primates, *in vivo* study of HCV infection has been hampered since its discovery more than 20 years ago. The chimpanzee remains the most physiological model to study the innate and adaptive immune responses, but its use is ethically difficult and is now very restricted and regulated. The development of a small animal model that allows robust HCV infection has been achieved using chimeric liver immunodeficient mice, which are therefore not suitable for studying the adaptive immune responses. Nevertheless, these models allowed to go deeply in the comprehension of virus-host interactions and to assess different therapeutic approaches. The immunocompetent mouse models that were recently established by genetic humanization have shown an interesting improvement concerning the study of the immune responses but are still limited by the absence of the complete robust life cycle of the virus. In this review, we will focus on the relevant available animal models of HCV infection and their usefulness for deciphering the HCV life cycle and virus-induced liver disease, as well as for the development and evaluation of new therapeutics. We will also discuss the perspectives on future immunocompetent mouse models and the hurdles to their development.

## INTRODUCTION

Hepatitis C virus (HCV) is a small enveloped positive sense single-stranded RNA virus from the Flaviviridae family (Lindenbach et al., 2007). HCV is one of the main causative agents of liver disease worldwide and a major problem of public health with approximately 130 million chronically infected people and more than 350,000 deaths each year. Moreover, the World Health Organization estimates that three to four million people are newly infected every year. No vaccine is available so far, and the current standard of care combination treatment of interferon-alfa (IFNα) and ribavirin is not effective against all HCV genotypes and often not well tolerated by the patients (Poordad and Dieterich, 2012). The recently developed direct-acting antivirals (DAAs) have significantly improved the treatment of chronic HCV infection (Poordad and Dieterich, 2012) but can lead to the selection of DAA-resistant viral variants (Pawlotsky, 2011; Aloia et al., 2012). Thus, despite the advances made in recent years, HCV infection remains a global health problem. Furthermore, a vaccine is not available. Research efforts are thus still to be continued in order to decipher the details of the viral life cycle within its host and to propose new therapeutic alternatives to improve patient care.

Improved understanding of the phenomena related to the interaction of the virus with its host, in its entirety, relies on the use of a model allowing the study of the whole viral life cycle as well as the host responses against the virus, especially the immune responses. Only the use of a living organism allows to achieve this goal. Chimpanzees are the only animals reliably supporting HCV infection and allowing the study of anti-viral immune responses even though they do not develop fibrosis and cirrhosis and very rarely hepatocellular carcinoma (HCC; Muchmore et al., 1988; Lanford et al., 2001). However, the use of chimpanzees in medical research is ethically very controversial and increasingly limited (Harrington, 2012). To overcome this hurdle, small animal models have been developed during the last decade. Mouse models are of particular interest since mice are easy to breed and handle and can be genetically modified.

In this review, we will discuss the advantages and limitations of the different animal models regarding their potential in leading to a better comprehension of virus-host interactions and HCV pathogenesis, as well as their utility for preclinical evaluations prior translation to clinical trials in human.

## NATURALLY HCV PERMISSIVE ANIMAL MODELS

Highly genetically HCV-related viruses from the hepacivirus gender can infect new world primates (Muerhoff et al., 1995; Simons et al., 1995), dogs (Kapoor et al., 2011), horses (Burbelo et al., 2012; Lyons et al., 2012) and bats (Quan et al., 2013). But so far, HCV was only found to infect few species other than human (reviewed in Simmonds, 2013), chimpanzees and non-rodent small mammal northern treeshrew (*Tupaia belangeri*).

### CHIMPANZEES

Chimpanzees are tightly linked to the history of HCV discovery (Houghton, 2009). These animals can be chronically infected with the virus, using various sources as inoculum. Although the clinical course of HCV infection in chimps and humans is not identical (Bukh, 2004), chimp studies have greatly contributed to our understanding of innate and adaptive immune responses in the course of HCV infection (Bowen and Walker, 2005; Rehermann, 2009). A large body of evidence indicates that T cell responses play a major role in viral clearance as well as protection from HCV infection (Neumann-Haefelin and Thimme, 2013). Indeed, memory T cells as well as the activation of intra-hepatic natural killer (NK) cells and type I/II interferon production were demonstrated to prevent HCV re-infection in chimpanzees (Nascimbeni et al., 2003; Barth et al., 2011). Moreover, neutralizing antibodies may also contribute to protection from HCV infection in these animals.

Chimpanzees have also been very valuable for the evaluation of various antivirals and to date, chimpanzees are the only animals permitting extensive evaluation of the efficacy of potential vaccines against HCV (Houghton, 2011). It has especially been shown that therapeutic vaccines including structural proteins are better T cell stimulators than vaccines where only non-structural proteins are present (Dahari et al., 2010). But so far, it was not possible to clearly identify a safe and effective vaccine for humans (Bailey, 2010).

Despite the fact that studies of HCV infection in chimpanzees have greatly advanced our understanding of the immune responses that are required to efficiently clear viral infection, several limitations of this model have to be pointed out (**Table 1**). Indeed, data from chimpanzee studies are highly variable and difficult to interpret mainly because of the biological variability between individual animals and the small animal cohorts. Moreover, chronic infection appears in only 30–40% of infected animals while it can reach 85% in humans (Lanford et al., 2001). Furthermore, chronically infected chimpanzees do not readily develop cirrhosis or fibrosis and have much milder symptoms (Bukh et al., 2001). It is worth noting that HCC is only rarely observed in chimpanzees (Muchmore et al., 1988; Lanford et al., 2001). Finally, the limited availability, the cost for acquiring and maintaining animals as well as ethic considerations have also been major drawbacks to the use of this animal model. Indeed, the use of chimpanzees for biomedical and behavioural research is now legally forbidden in Europe since the new European Directive 2010/63 and has been recently banned by the NIH (Harrington, 2012) following the recommendations of the Institute of Medicine (Altevogt et al., 2011).

**Table 1:** Current animal models for the study of HCV infection.

	Chimpanzee	Tree shrew	uPA-SCID	FRG	AFC8-huHSC/Hep	Rosa26-FLuc	Rat
	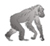	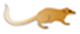	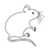	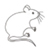	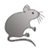	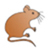	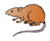
**HCV entry**	Yes	Yes	Yes	Yes	Yes	Yes	Yes	
**HCV production**	Yes	Yes	Yes	Yes	No	No	Yes	
**Viremia**	Weaker than in humans	Low	High	High	No	No	Low	
**HCV pathogenesis**	Milder than in humans, HCC?	Hepatitis, fibrosis, cirrhosis	No	No	Fibrosis	N/A	Hepatitis	
**Immune response**	Yes	Yes	No	No	Yes	Yes	No	
**MHC match Immune system/ hepatocytes**	Yes	Yes	N/A	N/A	Yes	Yes	No	
**MHC match Immune system/host**	Yes	Yes	N/A	N/A	No	Yes	Yes	
**Vaccine development**	Yes	Possible	No	No	Yes	No	No	

*HCC, hepatocellular carcinoma; N/A, not applicable.*

#### Tupaia belangeri

*Tupaia belangeri*, or Northern treeshrew, is a non-rodent small mammal susceptible to HCV infection (Xie et al., 1998; Xu et al., 2007; Amako et al., 2010). HCV entry factors CD81, scavenger receptor class B type I (SR-BI), claudin-1 (CLDN1) and occludin (OCLN) from tupaia origin have been shown to promote entry of HCV pseudoparticles or cell culture-derived HCV (HCVcc) into human or mouse cells engineered to express these host factors and primary tupaia hepatocytes are able to support HCV infection (Zhao et al., 2002; Barth et al., 2005; Tong et al., 2011). This model has recently been used to perform a metabolomic analysis upon HCV infection (Sun et al., 2013). In this study, the authors performed comprehensive metabolomics profiling and pathway analysis of large biological data sets in order to identify signaling pathways associated with HCV infection, but the HCV infection level of the animals was not clearly stated. Another study assessed the protective effect of xanthohumol, a main prenylated chalcone from hops, on HCV-induced hepatitis (Yang et al., 2013). Even though the authors were able to detect liver injury and HCV Core protein expression in the liver of HCV inoculated animals, neither serum HCV RNA nor anti-HCV antibodies could be detected. Indeed, despite the development of chronic liver disease in some animals, the infection rate of tupaia is weak and viremia appears to be low and rarely sustained (Xu et al., 2007; Amako et al., 2010; **Table 1**). Moreover, the limited availability of these animals, their cost of housing and the absence of tupaia-specific reagents to assess HCV-host interactions in this model still limit the use of tupaia for the study of HCV pathogenesis and vaccine design. The development of tupaia-adapted viruses may be one strategy to make this model more efficient and robust.

## RODENT MODELS OF HCV INFECTION

Mice and rats are naturally resistant to HCV infection as rodent hepatocytes do not support HCV entry and replication (Ploss et al., 2009; Dorner et al., 2011). It is worth noting that in contrast to the early steps of the viral life cycle, HCV assembly and release do not appear to be restricted in mouse cells (Long et al., 2011; Vogt et al., 2013). With the advent of transgenic technology different mouse models for the study of HCV infection could be engineered. Several transgenic mice carrying different parts of the HCV genome were the first available mouse models to study HCV-host interactions (reviewed in Lerat et al., 2011; Billerbeck et al., 2013). These mice display liver pathologies mimicking human disease, principally steatosis and primary liver cancer. However, in contrast to the human setting, as the mouse immune system tolerates the transgenically expressed viral proteins, liver pathogenesis establishes in the absence of local inflammation. Moreover, the absence of active HCV RNA replication in these mice precludes the study of HCV infection. Research efforts thus further focused on developing rodent models supporting productive HCV infection mainly by humanizing mice or rats to render them permissive to HCV. Humanization can be achieved by two different strategies: (i) xenografting human cells or (ii) transgenesis.

### THE uPA-SCID MOUSE MODEL

These immunodeficient (SCID) mice with hepatocyte-lethal phenotype due to the overexpression of the urokinase-type plasminogen activator (uPA) transgene in their liver can be efficiently engrafted with primary human hepatocytes in order to initiate infection with HCV (**Table 1**). The uPA-SCID mouse model was for the first time described more than 10 years ago (Mercer et al., 2001). It has been shown that the liver of these chimeric mice can be nearly completely repopulated by the transplanted human hepatocytes (Tateno et al., 2004). Such human liver chimeric mice then become susceptible to HCV infection. Following viral inoculation, HCV titers of more than 10^7^ IU/mL can been observed (Hiraga et al., 2007; Vanwolleghem et al., 2010) and viral infection can be sustained up to 10 months (Mercer et al., 2001). As for chimpanzees, in addition to HCV-positive patient-derived serum of different genotypes (Mercer et al., 2001; Hsu et al., 2003; Bukh et al., 2010), HCVcc may also be used to inoculate these mice (Lindenbach et al., 2006; Akazawa et al., 2013) and this thus enables researchers to study a wide variety of different inocula.

The uPA-SCID model has been extensively used to evaluate different strategies to prevent or treat HCV infection. Targeting cell entry of the virus is one of these approaches. HCV entry is a crucial step to establish infection and can be blocked by using neutralizing antibodies binding to the virions or by monoclonal antibodies (mAbs) targeting host entry factors. Studies using neutralizing antibodies purified from blood of a genotype 1a infected patient demonstrated the efficacy of this approach to inhibit viral infection with homologous and heterologous HCV strains (Vanwolleghem et al., 2008; Meuleman et al., 2011b). Another study assessed the potential of neutralizing mAbs from a phage display library constructed from the bone marrow mononuclear cell RNA of a chronically infected patient (Law et al., 2008). These antibodies have been shown to bind to the HCV envelope glycoprotein E2 and to exhibit broadly cross-neutralizing activity against heterologous HCV quasispecies in the uPA-SCID model. In addition to antibodies targeting the virus, HCV entry can also been inhibited by targeting host factors essential for this process (Zeisel et al., 2013). MAbs against the HCV entry factors CD81 (Meuleman et al., 2008) and SR-BI (Lacek et al., 2012; Meuleman et al., 2012) have been successfully tested in the chimeric uPA-SCID mouse model and were proven to be efficient at inhibiting HCV infection in challenge studies with different genotypes. Moreover, chimeric-liver mice that were already infected for 3 days could still be efficiently treated with five injections of 400 μg of anti-SR-BI mAb (Lacek et al., 2012; Meuleman et al., 2012). The uPA-SCID model has also been successfully used to assess the efficacy of small molecule drugs (Lupberger et al., 2011; Sainz et al., 2012) and various other molecules targeting host entry factors (Matsumura et al., 2009; Meuleman et al., 2011a). Indeed, administration of the clinically approved drug erlotinib, which specifically targets the tyrosine kinase activity of epidermal growth factor receptor (EGFR), significantly delayed the kinetics of HCV infection with a genotype 2a infectious serum (Lupberger et al., 2011). Another study showed that pretreatment with ezetimibe, an antagonist of the HCV co-entry factor Niemann-Pick C1-like1 (NPC1L1), could prevent infection of some chimeric mice infected with a genotype 1b virus (Sainz et al., 2012).

Moreover, the uPA-SCID mouse model has also been efficiently used to assess the efficacy of recently developed DAAs specifically targeting HCV encoded proteins required for viral replication. One of the first HCV protease inhibitor, BILN 2061, has been evaluated by Vanwolleghem et al. (2007) in this mouse model. Although the authors could see a very rapid viral load decline of about 2.5 log_10_ after a 4-day treatment, they also observed a deteriorating effect of this compound on the mouse cardiomyocytes. This latter observation confirmed results already seen in rhesus monkeys where BILN 2061 also induced cardiotoxicity (Reiser et al., 2005). Of note, the clinical development of BILN 2061 was halted due to these toxic effects. More recently, the clinically licensed NS3-4A protease inhibitor telaprevir has been evaluated in this model, alone (Kamiya et al., 2010) or in combination with the NS5B inhibitor MK-0608 (Ohara et al., 2011). Furthermore, a study by Shi et al. (2013) evaluated the antiviral activity of different combinations of DAAs against genotype 1b, 2a, and 2b in these mice. The authors assessed the effect of the NS3 protease inhibitor BMS-605339, the NS5A inhibitor BMS-788329 and the NS5B non-nucleoside analog inhibitor BMS-821095 and showed that different combination therapies were very efficient against genotype 1b virus but not against genotype 2a or 2b strains. Quasispecies population before and after treatment as well as selection of mutations leading to the appearance of resistant variants were also analyzed by ultra deep sequencing in these animals (Shi et al., 2013). Furthermore, several other compounds with an effect on viral replication have also been tested with success as monotherapy against HCV infection (Nakagawa et al., 2007; Kneteman et al., 2009; Narjes et al., 2011).

The uPA-SCID mouse model has further been used to study the relevance of the genetic polymorphism near the interleukin-28B (IL-28B) region described to be associated with a better response to IFNα and ribavirin in patients (Ge et al., 2009; Suppiah et al., 2009; Tanaka et al., 2009). In this study, Watanabe et al. (2012) showed that there was no significant difference in response to IFNα treatment in mice with human hepatocytes with different types of polymorphism indicating that the effect observed in patients was attributable to their immune responses, since these mice do not have an adaptive immune system.

Although the uPA-SCID mouse model has proven its usefulness and has become relevant for the preclinical evaluation of novel antiviral compounds, this model has several limitations. Indeed, these mice are very fragile and have to be engrafted within the first weeks of life given that these animals are born with a hepatocyte-lethal phenotype (Meuleman et al., 2005; Vanwolleghem et al., 2010). Moreover, the uPA transgene can be deleted in some mice leading to the restoration of a wild-type phenotype and thus loss of the human hepatocyte graft (Sandgren et al., 1991). Another drawback of this model is the absence of a functional adaptive immune system. Due to the severe combined immunodeficiency background, these mice are deficient in functional mature T and B cells. Therefore, they cannot be used for the study of adaptive immune responses or for the evaluation of vaccines. Nevertheless, this model is not devoid of innate immunity and has been used to decipher HCV specific innate immune responses (Walters et al., 2006).

### The Fah^-/-^ Rag2^-/-^γ-c^-/-^ (FRG) MOUSE MODEL

In order to overcome some of the limitations of the uPA-SCID mouse model, another model has been developed using immunodeficient mice with genetic alterations leading to a hepatocyte-lethal phenotype. These “FRG” mice are deficient for the Rag2 recombinase and the common γ-chain of the interleukin receptors, leading to a more profound immunodeficiency (Goldman et al., 1998; Mazurier et al., 1999). Moreover, they are deficient for the tyrosine catabolic enzyme fumarylacetoacetate hydrolase (Fah), which leads to liver degeneration (Grompe et al., 1993, 1995; Overturf et al., 1996). In this model, liver degeneration can be prevented as long as the 2-(2-nitro-4-trifluoromethylbenzoyl)-1,3-cyclohexanedione (NTBC) drug is provided to the mice. Thus, the time of transplantation of human hepatocytes is easier to control than in the uPA-SCID mouse model since it can be done at anytime in adult mice upon drug withdrawal. Moreover, in contrast to the uPA-SCID mouse model, spontaneous reversion of the hepatocyte-lethal phenotype does not occur since there is a full deletion within the Fah encoding gene (Grompe et al., 1993).

Upon NTBC withdrawal, FRG mice can be efficiently transplanted with human hepatocytes to obtain liver repopulation (Azuma et al., 2007; Bissig et al., 2007). Moreover, Bissig et al. (2010) successfully infected these transplanted mice with a genotype 2a HCV JFH-1 strain, a clinical isolate of HCV genotype 1a and chimeric genotype 1a/2a and 1b/2a viruses. Up to now, this study is the only one reporting HCV infection in the FRG model. Although this chimeric liver mouse model has so far been less extensively used than the uPA-SCID model, it should be as efficient to allow preclinical evaluation of antiviral compounds (**Table 1**) and several reports using this model are expected within the next years.

Like the uPA-SCID mouse model, the FRG model suffers from a lack of an immune system. Due to the Rag2 and γ-c deficiencies, these mice do not harbor T-, B- and NK-cells. These animals are therefore not more suitable than the uPA-SCID mice for the study of HCV immunopathogenesis and for the development and evaluation of vaccines.

### AFC8-huHSC/Hep MICE

Recently, another model of HCV permissive mice has been developed to overcome the lack of immunity intrinsic to the uPA-SCID and FRG models. This model is based on immunodeficient Balb/c Rag2^-/-^γ-c^-/-^ (BRG) mice that have been genetically modified to overexpress in the mouse liver a fusion protein of the FK506 binding protein (FKPB) and caspase-8 under the albumin promoter (AFC8; Washburn et al., 2011). Injection of AP20187 induces the homodimerization of the caspase-8 active domain leading to a suicidal activity of this enzyme and thus death of mouse hepatocytes (Pajvani et al., 2005). This induced hepatodeficiency improves the engraftment of human hepatocytes. These transgenic mice can therefore be transplanted, within the 5 first days of life, with human hepatocyte progenitor cells and CD34^+^ hematopoietic stem cells derived from the same fetal liver allowing human leukocyte antigen (HLA) matching between these cells. Following injection of AP20187, the authors demonstrated development of human immune cells and human hepatocytes in these mice. About 50% of these AFC8-huHSC/Hep mice were subsequently effectively infected with patient serum-derived HCV of genotype 1a but HCV RNA could only be demonstrated in the liver of these animals. No HCV RNA was detected in the blood of infected mice. Despite this absence of viremia, the authors observed human immune cell infiltration in the liver of HCV^+^ mice as well as HCV-specific CD4 and CD8 T cell responses. Specific B cell responses could not be observed in these animals. Interestingly, half of these HCV^+^ mice developed severe portal fibrosis with numerous septa. This was the first report on a small animal model of HCV infection exhibiting development of HCV-specific adaptive immune responses and virally induced immunopathogenesis (**Table 1**).

Although these mice represent a first breakthrough for the development of a fully immunocompetent mouse model for the study of HCV infection, the absence of serum HCV particles and the lack of fully functional B cells are important issues for the evaluation of potential antiviral drugs and vaccines. Furthermore, the education of the immune system from human HSC is done on the murine major histocompatibility complex (MHC). It is not yet clear whether the selection of human immune cells on mouse MHC will allow proper recognition of HCV infected HLA expressing human hepatocytes and be comparable with the human setting.

### Rosa26-Fluc HCV ENTRY FACTOR HUMANIZED MICE

**FIGURE 1:**
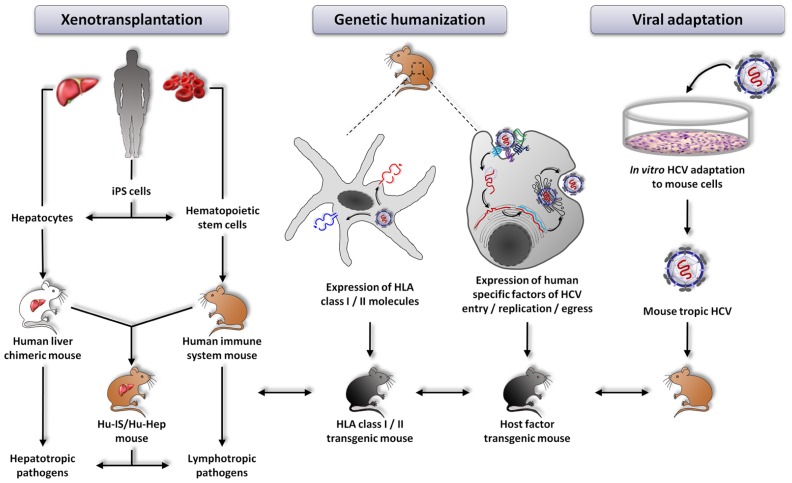
**Currently explored approaches aiming at developing new animal models for the study of hepatitis C virus infection**. From left to right: xenotransplantation of human cells, genetic humanization by expression of defined human specific factors, viral adaptation of HCV to mouse cells. HLA transgenic mice can be crossed with the different models in order to improve immune responses.

Another model of immunocompetent mice permissive to HCV infection has been developed shortly after the AFC8-huHSC/Hep. In contrast to the previous model, these mice have a fully functional murine immune system and liver. To overcome the species-specific restriction of viral entry and to achieve HCV susceptibility of the mouse liver, the authors targeted the mouse hepatocytes *in vivo* with adenoviral vectors to express human HCV entry factors CD81, SR-BI, CLDN1, and OCLN (Dorner et al., 2011; **Figure 1**). About 5% of the murine hepatocytes expressed all four human entry factors upon adenoviral transduction. The Rosa26-Fluc background of these mice allows to detect viral entry by *in vivo* bioluminescence. Indeed, as HCV does not efficiently replicate in mouse cells (Zhu et al., 2003; Uprichard et al., 2006; Lin et al., 2010), Dorner et al. (2011) engineered the virus to express the Cre recombinase (HCV-CRE). This latter, once expressed in the mouse liver, leads to the activation of a loxP-flanked luciferase reporter in the genome of the Rosa26-Fluc mice (Safran et al., 2003). The emitted photons then reflect viral entry into hepatocytes. This model was successfully used to study for the first time entry of HCVcc chimeras of different genotypes into mouse hepatocytes *in vivo*.

The authors also assessed the efficacy of anti-CD81 and anti-E2 antibodies on HCV entry as well as a prophylactic vaccine based on recombinant vaccinia virus encoding HCV proteins from Core to NS2 of genotype 1a. With this vaccine approach, robust titers of anti-E2 antibodies and a decreased susceptibility to heterologous challenge with HCV-CRE harboring structural proteins of genotypes 1b, 2a, or 4a was observed. This is the first report of the usage of a fully immunocompetent small animal model for the study of HCV infection and, so far, this model is the only available mouse model for combined immunization and challenge studies. This is also the only mouse model with a perfect MHC matching between the immune system and infected hepatocytes, which would allow a better understanding of the entire immune responses induced by the virus (**Table 1**).

Although conceptually markedly advancing the field, the humanized Rosa26-Fluc model still has its limitations. First, there is no virus production by the infected mouse hepatocytes due to inefficient viral replication in these cells. This makes this model unsuitable for the evaluation of DAAs or antivirals targeting the assembly and egress steps of the viral life cycle. Moreover, the usage of adenoviral vectors to introduce the human entry factors into mouse hepatocytes leads to the induction of an immune response against the vector. This renders the study of HCV-specific immune responses difficult since the induction of interferon stimulated genes (ISGs) and a rapid NK cell-mediated innate immunity lead to the loss of adenovirus-transduced hepatocytes and therefore to the loss of HCV infected cells. Thus, it is not possible to study HCV-induced immunopathogenesis in this model. This model is currently being extended by developing transgenic mice stably expressing the human HCV entry factors and ultimately further engineering the mouse hepatocytes to support HCV replication. A first transgenic mouse expressing human CD81, SR-BI, CLDN1, and OCLN did not appear to be susceptible to HCV entry (Hikosaka et al., 2011) but technical limitations may have precluded detection of viral entry in this study. Follow-up studies using human entry factor transgenic mice are thus awaited to assess the potential of such mice for further studying HCV pathogenesis.

### IMMUNOCOMPETENT RAT MODEL

Another immunocompetent small animal model susceptible to HCV infection was generated using rats tolerant to human hepatocytes (Ouyang et al., 2001). Fetal rats were intraperitoneally injected with human hepatoma Huh7 cells between 15 and 17 days of gestation in order to induce a specific tolerance of the rat immune system toward these cells. This tolerance allows the transplantation of Huh7 cells via an intrasplenic injection in newborn rats, within 24 h of birth. This results in survival of these cells without rejection by the rat immune system as demonstrated by human albumin detection in the liver as well as in the serum of the tolerized transplanted rats (Ouyang et al., 2001). These rats may then be inoculated with serum-derived HCV of genotype 1 one week after transplantation and are able to develop transient viremia as shown by an HCV viral load of 2 × 10^4^ copies/mL by week 12 (Wu et al., 2005). Infected animals where characterized by inflammation of the liver as demonstrated by elevated serum alanine aminotransferase with a peak at week 13 as well as by mononuclear cell infiltration in the liver (**Table 1**).

Unfortunately, the set-up of this model is difficult since the intraperitoneal injection in fetal rats is challenging. Moreover, this model is limited by the fact that the transplanted cells are not primary human hepatocytes but a human hepatoma cell line. Furthermore, the number of transplanted cells is low and viremia remains weak, around 2 × 10^4^ copies/mL, in comparison to viral loads observed in HCV-infected patients or human liver chimeric mice. Despite the fact that this animal model is immunocompetent and can be infected with HCV, the mismatch between human HLA and rat MHC prevents the study of adaptive immune responses against infected hepatoma cells. It is worth noting that this model has not been extensively used so far and only one publication reported its usage (Wu et al., 2005). Further reports would thus be useful in order to compare the potential of this model to the potential of the different mouse models to study HCV pathogenesis and assess antiviral strategies.

## FUTURE DIRECTIONS

In the past years, much progress has been made in developing novel animal models for the study of HCV-host interactions. The major drawbacks of the different small animal models described so far are (i) absent or not fully functional adaptive immunity, (ii) low-level viral replication and absent viremia and consequently (iii) absent or low fibrosis and no cirrhosis. The uPA-SCID and FRG models both lack T and B cells. The AFC8-huHSC/Hep model is characterized by a human immune system educated on murine MHC molecules and absent viremia. The Rosa26-Fluc model has matched murine immune cells and hepatocytes but these latter are unable to efficiently replicate the viral genome and to egress the virus. Finally, the immune system of the immunocompetent rat model does not match the HLA molecules at the surface of the transplanted human hepatoma cells. Thus, additional immunocompetent mouse models need to be developed to study different aspects of HCV pathogenesis in the context of robust viral replication. So, ultimately, what would be the best model to study HCV infection? Different lines of development are currently pursued: (i) adapting the virus to mice to allow the virus to accomplish its entire life cycle in mouse hepatocytes, (ii) further humanizing mice to render these animals permissive to HCV and (iii) combining these two approaches (**Figure 1**).

### HCV-ADAPTED IMMUNOCOMPETENT MOUSE MODEL

Given that mouse hepatocytes are resistant to HCV infection, a possibility to overcome this species-specific restriction of viral infection without genetically engineering the host is to adapt the virus to mouse cells, i.e., develop a virus able to complete its whole life cycle - entry, translation, replication, egress – in normal mouse hepatocytes (**Figure 1**). Bitzegeio et al. (2010) have explored this trail by the selection of a HCV Jc1-derived mutant (genotype 2a) adapted to the murine entry factor CD81. In contrast to wild-type Jc1, this HCV mutant is able to enter cells expressing mouse CD81. However, despite efficiently entering mouse cells, this selected HCV strain is unable to replicate in mouse hepatocytes, indicating that host factors limit productive infection downstream of virus cell entry (Bitzegeio et al., 2010). Several lines of evidence indicate that innate immune responses interfere with HCV replication and although HCV has evolved strategies to evade innate immunity in human cells, this may be less efficient in mouse cells (Lin et al., 2010; Billerbeck et al., 2013; Schoggins and Rice, 2013; Vogt et al., 2013). So potentially the selection of a mouse innate immune response-insensitive HCV strain might overcome the limitations imposed by mouse hepatocytes and lead to the development of viremia in immunocompetent mice. However, even though such a model might be achievable, it will remain to be determined to which extent it would be comparable to HCV infection in humans.

In order to mimic more precisely the human immune response, it might be of interest to use HLA expressing mice (Pajot et al., 2004), in line with a recent study where hepatitis B virus genome was introduced into the liver of HLA-A2/DR1 mice (Dion et al., 2013). However, the main limitation of such an approach is the fact that it will be very difficult to mimic the variability of HLA combinations present in humans. Thus, efforts should focus on defined HLA transgene combinations. Nevertheless, there is no certainty that the mouse immune responses developed against the virus would be comparable to those observed in human (Mestas and Hughes, 2004).

Therefore, another strategy to generate a fully immunocompetent mouse model for the study of HCV infection is to focus on the development of mice with humanized immune system and humanized liver (hu-IS/hu-Hep mice).

### HUMAN IMMUNE SYSTEM – HUMAN HEPATOCYTE CHIMERIC MOUSE MODEL

The optimal way to assess the role of the immune system in response to HCV infection and to explore virus-induced immunopathogenesis in a setting comparable to humans would be to use mice harboring both human immune cells and human hepatocytes (**Figure 1**). The study by Washburn et al. (2011) described above paved the road to the development of such an animal model. However, the selection of human immune cells on murine MHC instead of HLA molecules may have precluded the development of very efficient T and B cell responses. An alternative approach would thus be to use hepatodeficient and immunodeficient HLA-expressing mice in order to allow the engraftment of HLA matched human hematopoietic stem cells (hHSC) and human hepatocytes. The selection of immune cells on HLA molecules and the recognition of the hepatocytes as being from self might give a better view of the immune response against HCV and allow the design of efficient vaccines and new therapeutic products.

Unfortunately, the development of all hematopoietic compartments from hHSC in mice is not efficient because of the inability of several mouse cytokines to stimulate human cells (Manz, 2007; Legrand et al., 2009). In order to ameliorate the immune reconstitution from hHSC, several approaches have been attempted using injection of exogenous recombinant cytokines or by creating transgenic or knock-in mice (reviewed in Willinger et al., 2011). However, to achieve a complete human immune reconstitution, several different human cytokines and growth factors would be necessary. Moreover, several mouse strains with different genetic backgrounds reject transplanted human cells because of the inefficient interaction between human CD47 and the mouse signal-regulatory protein alpha (SIRPα) expressed on macrophages (Takenaka et al., 2007). This inability of human CD47 to interact with mouse SIRPα leads to activation of the phagocytic activity of macrophages (Takizawa and Manz, 2007). In line with this observation, it has been shown that human progenitor cells expressing mouse CD47 can efficiently be engrafted in BRG mice leading to a better homeostasis of T- and NK-cells in lymphoid organs (Legrand et al., 2011). Similarly, the same BRG background, which is one of the most efficient to allow hHSC transplantation, has been used to create human SIRPα transgenic mice in order to improve engraftment of hHSC (Strowig et al., 2011). In order to avoid genetic manipulations and selection procedures of the cells to be transplanted, which can be in limited number at the time of the graft, other genetic backgrounds have been investigated for their efficiency to accept xenogenic transplantations. This lead to the selection of the non-obese diabetic (NOD) background which appears to be the best recipient for hHSC transplantation. Indeed, this strain exhibits a polymorphism of the gene encoding SIRPα, allowing a more efficient binding to human CD47 (Takenaka et al., 2007; Takizawa and Manz, 2007; Yamauchi et al., 2013). Therefore, the NOD background may be more suitable to introduce xenogenic cells without the need of genetic modification in order to achieve high degree of chimerism (reviewed in Ito et al., 2008). The recent advances in the field of induced pluripotent stem cells (iPS) may allow in the future to develop a mouse model engrafted with iPS-derived hepatocytes and HSC from the same donor (Espejel et al., 2010; Huang et al., 2011; Liu et al., 2011; Sekiya and Suzuki, 2011; Schwartz et al., 2012; Wu et al., 2012). This might overcome the issues of HLA-matching between the immune system and hepatocytes but will not resolve HLA matching with the host. However, this strategy using iPS cells, which could be available in large amount, would be easier to implement than the use of hHSC and fetal hepatocytes which are more complicated to access due to ethical reasons.

The hu-IS/hu-Hep mouse model would certainly be the best model to assess the immune responses against HCV, to decipher more deeply the immunopathogenesis developed during chronic infection, to explore HCV-host interactions during acute infection and to unravel the mechanisms leading to virus eradication as well as to develop vaccines and new therapeutic approaches.

### GENETICALLY HUMANIZED MOUSE MODEL

 Another explored approach in order to develop an immunocompetent mouse model of HCV infection relies on genetic modifications of mice by introducing essential human specific factors for the viral life cycle (**Figure 1**). It has previously been shown that viral entry, the first step of HCV infection, requires the presence of at least two human cell surface factors, CD81 and OCLN (Ploss et al., 2009). The study by Dorner et al. (2011) described above has shown that adenoviral expression of human entry factors in mouse liver enables viral entry into murine hepatocytes *in vivo*. HCV RNA replication thus appears to be the next and last essential step to overcome in mouse cells in order to reconstitute the entire viral life cycle since mouse cells are able to support viral assembly and egress (Long et al., 2011). Indeed, it has been shown that viral RNA is translated in mouse cells but is unable to replicate efficiently (McCaffrey et al., 2002; Dorner et al., 2011). It is worth noting that several studies have shown that HCV replicons can replicate in murine cell lines (Zhu et al., 2003; Uprichard et al., 2006; Frentzen et al., 2011), indicating that there are no dominant murine inhibitory factors implied in the low replication of HCV in mouse cells and that murine orthologs of host factor required for viral replication are able to participate in the full life cycle of HCV. The activation of mouse innate immune responses may thus most likely be responsible for the limited HCV replication in mouse cells (Schoggins and Rice, 2013). Indeed, it has been shown that inactivation of several antiviral cellular molecules involved in innate immunity enhances HCV replication and allows HCV production by mouse cells (Chang et al., 2006; Lin et al., 2010; Aly et al., 2011; Vogt et al., 2013). It might thus be wise to ascertain *in vivo* the relevance of these findings and to generate a mouse model devoid of some of these innate immune pathways or knock-in mice expressing human orthologs of these innate immune mediators.

## CONCLUSIONS AND PERSPECTIVES

Since the development of the first small animal model of HCV infection – the uPA-SCID mouse model (Mercer et al., 2001), other increasingly sophisticated models emerged (Wu et al., 2005; Bissig et al., 2010; Dorner et al., 2011; Washburn et al., 2011). Each of these models allowed to significantly advance our understanding of defined aspects of HCV infection and HCV-host interactions and to pave the way for future animal models combining different characteristics and advantages of each model. Although the uPA-SCID mouse model has been most intensively used as a preclinical model in order to assess different classes of antivirals, none of the current models prevails over the others with respect to analysis of all aspects of viral infection (virus life cycle, immune response, pathogenesis, vaccine development…). The combination of different technologies and efforts will ultimately lead to the development of additional models better suited for the study of HCV immunopathogenesis and vaccine development. Given the natural history of HCV infection, requiring decades to evolve toward an HCC, one may consider that obtaining HCV-induced cirrhosis and HCC will be highly challenging in rodents, whose life expectancy is around 2 years. However, numerous models of HCC based on the transgenic expression of HCV proteins have been published (reviewed in Billerbeck et al., 2013), suggesting that HCV infection-induced HCC may be achievable, provided that sufficient host-responses are generated. As the chimpanzee model has to be abandoned in favor of small rodent models, the well known genetics of the mouse and the ease of modification of its genome should put this animal first in line to become the next gold standard for HCV research. Fully mouse or half human, the possibilities remain open. The complementarities of both approaches will raise new perspectives in the field of animal research for HCV and for the development of new therapeutic alternatives. The quest for the “holy Grail” is on, but the road is still long and full of pitfalls.

## Conflict of Interest Statement

The authors declare that the research was conducted in the absence of any commercial or financial relationships that could be construed as a potential conflict of interest.

## Abbreviations

HCV: hepatitis C virus
HCC: hepatocellular carcinoma
